# Delicate Test for Albumen

**Published:** 1880-07

**Authors:** 


### DELICATE TEST FOR ALBUMEN.

SIEBOLD has introduced a modification of the heat test which is adequate to the detection of albumen under conditions in which its presence might be completely overlooked. The following is the author's own account of the manner in which the test is to be applied: "Add solution of ammonia to the urine until just perceptibly alkaline; filter and add diluted acetic acid very cautiously until the urine acquires a faint acid reaction, avoiding the use of a single drop more than required. Now place equal quantities of this mixture in two test tubes of equal size, heat one of them to ebullition and compare it with the cold sample contained in the other test tube. The least turbidity is thus distinctly observed, and gives absolute proof of the presence of albumen."

